# Assessing family planning progress in exemplar countries: development of a conceptual framework and case study methodology

**DOI:** 10.1136/bmjgh-2024-018769

**Published:** 2026-06-09

**Authors:** Zahid Ali Memon, Hina Najmi, Abeer Mian, Sacha St-Onge Ahmad, Muhammad Islam, Kevin Samuel, Marleen Temmerman, Ali Mohammad Mir, Zeba Sathar, Zulfiqar A Bhutta

**Affiliations:** 1 Department of Community Health Sciences, The Aga Khan University, Karachi, Sindh, Pakistan; 2 Institute of Global Health and Development, The Aga Khan University, Karachi, Sindh, Pakistan; 3 Centre for Global Health and Child Health, The Hospital for Sick Children, Toronto, Ontario, Canada; 4 Centre of Excellence in Women and Child Health, The Aga Khan University Hospital Nairobi, Nairobi, Kenya; 5 Population Council Pakistan Office, Islamabad, Pakistan

**Keywords:** Global Health, Health services research, Study design

## Abstract

Family planning (FP) is a critical component of reproductive health, enabling individuals to plan the number and timing of children through various contraceptive methods.

We outline the conceptual framework and country case study methodology used by the Exemplars in Family Planning (EFP) project. This was based on the wider Exemplars in Global Health programme, which uses mixed-methods to examine determinants of FP progress in countries that exceed expectations for health outcomes. The EFP project integrates findings from case studies on six such countries, including quantitative analyses of trends and drivers of modern contraceptive prevalence, systematic reviews, policy, programme and financing assessments, and qualitative data collection with policymakers, providers and community actors. Data triangulation across these methods, complemented by consortium input and country workshops, enabled robust validation of findings and identification of cross-cutting themes. This approach produced a nuanced understanding of FP progress by capturing interactions between government programmes (demand and supply), service delivery mechanisms, subnational contexts and individual determinants. Quantitative decomposition and trend analyses quantified the contributions of population characteristics, behavioural factors and interventions, while qualitative and stakeholder data contextualised these findings within local sociocultural, health system and policy environments.

This multicountry, mixed-methods analysis led by local researchers and supported by a global consortium enhanced methodological rigour, contextualised findings and enabled cross-country learning on FP progress. It identified key cross-cutting drivers and offers evidence-informed guidance to improve contraceptive uptake and demand satisified, while highlighting the need for context-specific interventions and further longitudinal, multicountry research.

**SUMMARY POINTS**

- Despite progress, many low- and middle-income countries continue to face challenges such as limited access to contraception due to cultural barriers, inadequate infrastructure and health system constraints, hindering family planning efforts at a time when the global population continues to grow.
- We present an evidence-based conceptual framework for the Exemplars in Family Planning project, informing the design of methods, tools, data collection, analysis and triangulation for six country case studies.
- We employed a mixed-methods approach integrating quantitative, qualitative, policy, programmatic and financial data, along with systematic reviews of existing literature. This approach enabled triangulation of findings, validation of results and a nuanced understanding of changes in modern contraceptive prevlance rate, helping to identify the drivers of family planning progress and explain how and why change occurred in each country’s context.
- The mixed methods approach was strengthened through workshops and within-country synthesis, aligning data to shared frameworks to ensure consistency and coherence.

## Introduction

 Family planning (FP) enables individuals to make informed decisions about the number and spacing of their children through a range of contraceptive methods,^1^ broadly classified as traditional or modern. Traditional methods are typically defined as non-medical, behaviour-based practices rooted in cultural or community knowledge, whereas modern methods are generally defined as clinically tested, standardised and widely used due to proven effectiveness in preventing pregnancy.^2 3^ Significant progress has been made, with use among women of reproductive age rising from 55% in 1990 to 77% in 2022,^4^ and global fertility rates declining from 3.3 in the 1990s to 2.3 in 2021.^4 5^ This has improved maternal and child health by reducing unintended pregnancies, unsafe abortions and closely-spaced births.^6^ However, the global population continues to grow, projected to surpass 8.5 billion by 2030, primarily in low- and middle-income countries (LMICs).^7^ These regions continue to face persistent challenges, including inadequate infrastructure, cultural and religious barriers and limited access to modern contraception, which hinder the effectiveness of FP efforts.^5 8 9^ Globally, an estimated 218 million women in LMICs have an unmet need for modern contraception, leading to approximately 111 million unintended pregnancies and 35 million unsafe abortions annually.^10^ These outcomes drive maternal and infant morbidity and mortality, limit women’s educational and economic opportunities and strain health systems, slowing national development progress.^11^ This highlights the urgent need for sustained investment in expanding access to modern contraceptive methods.

Recent strategies used widely by LMICs to drive increases in modern contraceptive prevalence rate (mCPR) include advocacy campaigns on awareness and use of contraceptive methods among couples, community mobilisation efforts (involving religious leaders),^12^ media campaigns,^13 14^ door-to-door counselling^15^ and public-private partnerships.^16^Policy reforms in many sub-Saharan African countries have advanced gender equality and reproductive rights, improving outcomes.^8^ Many countries in Latin America,^17^ Southeast Asia and Africa experienced a considerable increase in contraceptive use in the early 1990s; however, more sustained progress between 1990 and 2020 was observed in settings with stronger political commitment and consistent investment in the social sector.^18^ At present (2025), the mCPR remains low in LMICs at 56%,^19^ indicating the need for continued efforts. Lessons on the way forward may be gleaned from countries that have made significant progress in recent years.

The Exemplars in Global Health (EGH) research programme is based on deriving insights from such ‘positive outliers’.^20^ In this paper, we present the steps taken to conduct the Exemplars in Family Planning (EFP) project. We separately report findings from those individual country case studies, as well as a cross country synthesis - both highlighting shared learning and insights into drivers of progress in FP uptake.

## Project governance

The EFP project operated through a coordinated structure that included a global consortium led by the Aga Khan University, an expert Technical Advisory Group (TAG), and in-country research partner per country (country selection methods detailed below). The project was funded by the Gates Foundation and received technical and coordination support from Gates Ventures.

### Consortium

The consortium comprised three cross-disciplinary research institutions from Pakistan and Canada that provided technical support and co-leadership for all project components. The team integrated expertise across qualitative research, advanced quantitative analyses and policy and programme evaluation to examine FP trends across diverse settings. This approach facilitated continuous exchange between global and in-country research teams, balancing analytical consistency with contextual adaptation. Through this, we generated actionable, policy-relevant evidence and were able to leverage cross-disciplinary collaboration to produce robust and applicable insights to programme and policy decision-making.

### Technical advisory group

The TAG consisted of ten experts from Asia, Africa, Latin America and North America, ensuring the incorporation of diverse regional perspectives and programmatic experience. Members included academics, researchers, policy analysts and representatives from development partners and donor agencies. Collectively, the group contributed a wide range of technical expertise spanning mixed-methods research, FP programming, maternal and child health, gender and social norms and equity-focused approaches.

Throughout the project cycle, the TAG contributed to strengthening methodological rigour, enhancing the quality of in-country evidence generated and guiding key strategic decisions. The group provided technical guidance on the conceptual framework, research design and case study methodologies, ensuring alignment with high-impact FP practices and contextual relevance for countries selected. TAG members also reviewed the selection of in-country research partners, assessed local technical capacity and advised on approaches to ensure robust and contextually grounded data collection.

As the research progressed, the TAG reviewed preliminary findings from country teams and provided detailed feedback to strengthen analytical rigour and improve cross-country comparability. In the final stages, the group validated the synthesis of findings across countries and recommended strategies for dissemination and cross-learning, helping translate the evidence into actionable policy and programmatic guidance.

### Countries onboarding

Alongside technical expertise in FP and Sexual and Reproductive Health (SRH), in-country research partners were selected for their experience with large-scale quantitative and qualitative research and for established networks with relevant national stakeholders, non-governmental organisations and government institutions. Partner responsibilities included obtaining national FP financing, policy and survey data, ensuring buy-in from key national stakeholders for the project’s findings through inception and dissemination meetings and conducting primary data collection at the country level.

## Methods

### Country selection

FP Exemplar countries were selected using a time-lag analysis approach. Simple linear regression was used to examine the relationship between the Human Development Index (HDI) and FP outcomes, including mCPR, demand satisfied for modern contraceptive methods and changes in these indicators since 2010 across different time periods. Countries whose observed values significantly exceeded predicted values for mCPR and demand satisfied at the 5% significance level were identified as ‘exemplary’ countries. Modelled annual estimates for mCPR and demand satisfied were obtained from the UN Population Division, while HDI data were sourced from the United Nations Development Programme. The analysis included 130 countries with available data on these indicators. In the second round of country selection, the residual significance threshold was relaxed to 30% to capture countries demonstrating progress beyond sub-Saharan Africa. This adjustment allowed for greater geographic representation, including countries from regions such as Latin America and Southeast Asia, by identifying countries where observed performance exceeded expected values at a 70% confidence level.^20^ The countries selected in round 1 were Senegal, Malawi and Kenya and in round 2 were the Lao People’s Democratic Republic (Lao PDR), Bolivia and Sierra Leone.

### Conceptual framework

**Figure 1:**
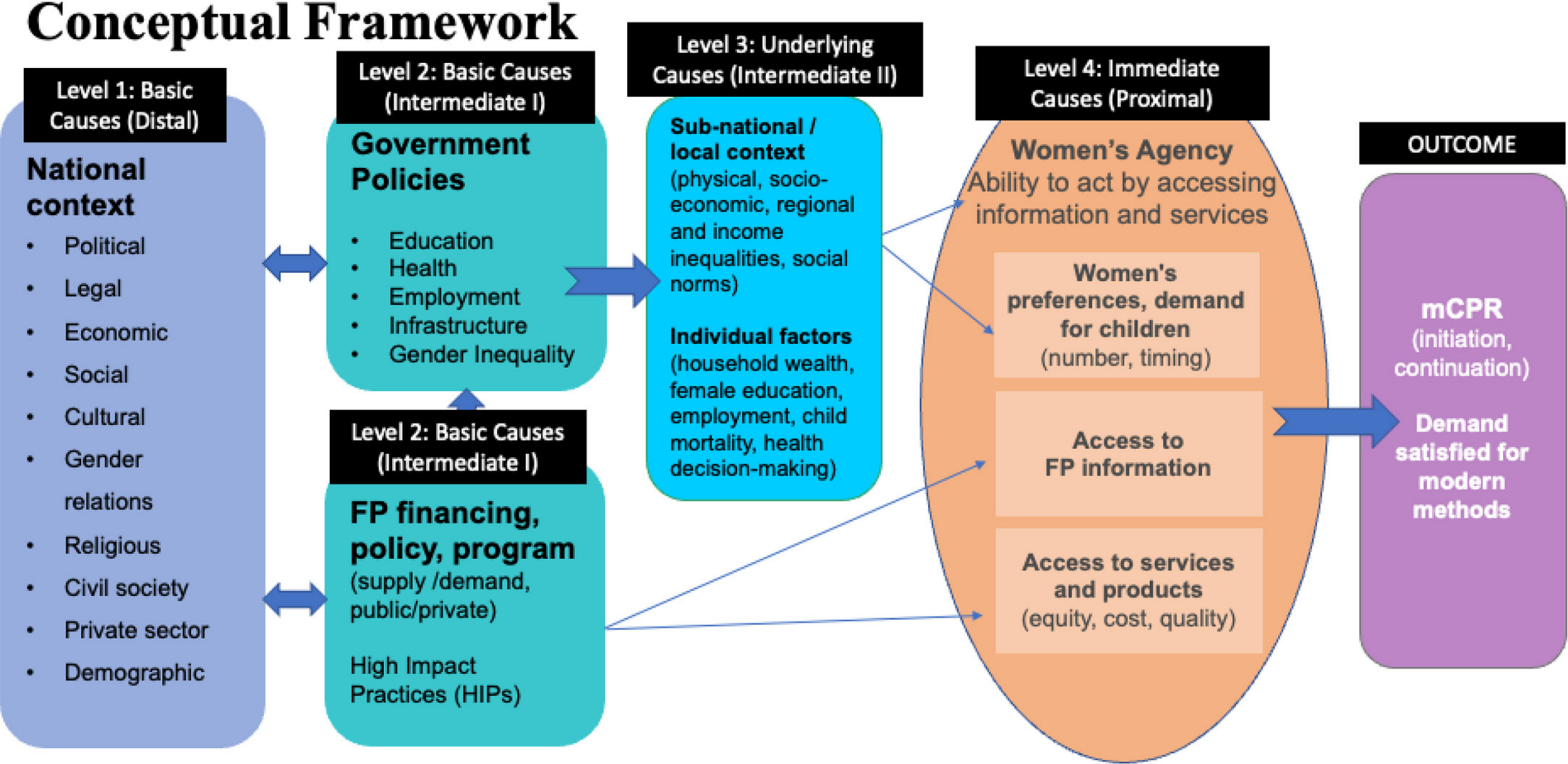
Conceptual framework for determinants of family planning outcomes. FP, family planning; mCPR, modern contraceptive prevalence rate.

A conceptual framework was designed to guide country case study methodologies and to provide a holistic understanding and approach for synthesis of evidence on mCPR, recognising the multisectoral nature of the issue and the complex interplay among distal, intermediate and proximal determinants (figure 1). The framework identifies key contributors to changes in mCPR and demand satisfaction within FP, drawing on global evidence. The approach aligns with the UNICEF Conceptual Framework on the Determinants of Maternal and Child Nutrition^21^ which emphasises the interaction among immediate, underlying and enabling determinants of population outcomes.

The framework operates at two levels. At the macro level, the framework examines national factors, including political, economic, social and cultural influences, that shape an enabling environment for FP. At the micro level, it incorporates subnational and local contexts, including regional disparities, income inequalities and social norms that directly affect perceptions and access to FP services. Central to the framework is the concept of women’s agency, emphasising the importance of empowering women with access to information, services and products aligned with their reproductive goals. All factors included in the framework have demonstrated significant associations with reproductive health and FP outcomes globally.

The framework provided a structured approach for examining policy environments, health system performance, socioeconomic and gender dynamics and service delivery pathways across countries, using mixed methods. Developed through iterative consultations with project researchers and guidance from the TAG, it enabled systematic cross-country comparison while maintaining contextual sensitivity.

The current framework represents an advancement to existing behavioural frameworks, including the Socio-ecological model,^22^ Health Belief Model^23^ and Theory of Planned Behavior^24^ which are central to explaining contraceptive decision-making because they highlight how individual perceptions, social norms and perceived agency shape health behaviours. However, these models primarily operate at individual and interpersonal levels and may not fully capture the broader policy, financing and health system dynamics that influence access and sustained FP use.

### Country case studies

**Figure 2:**
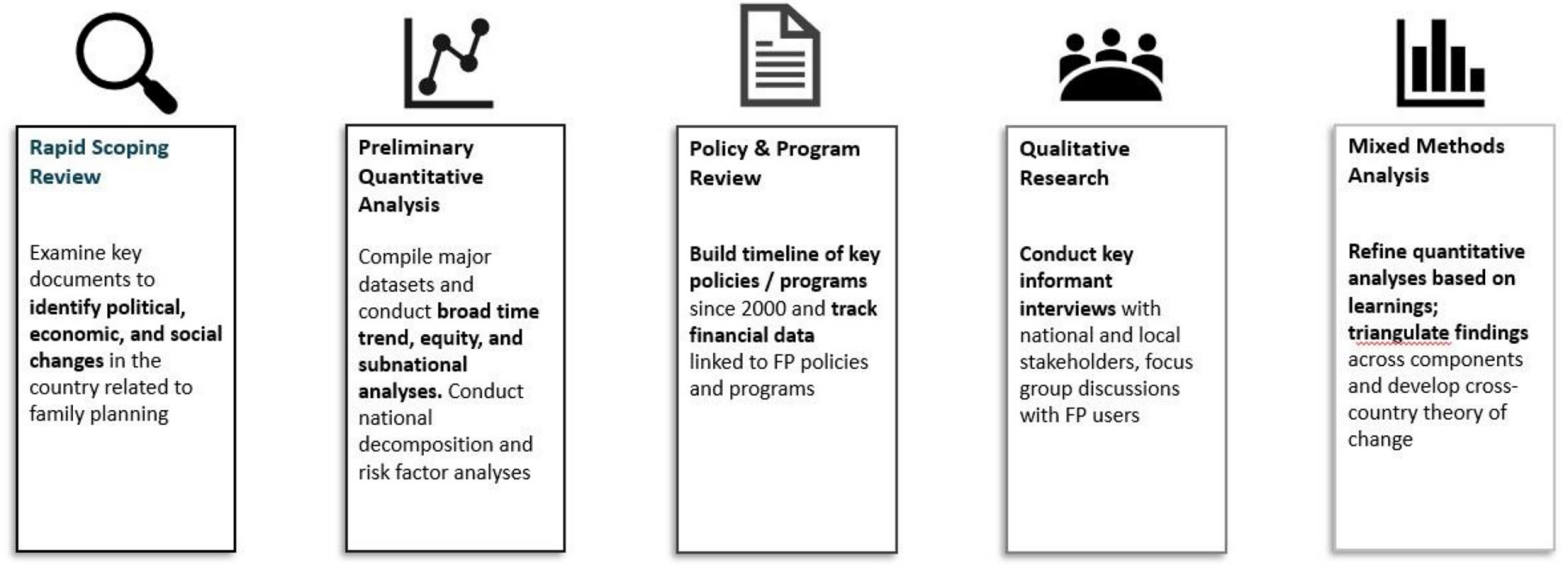
Overview of country case study methods. FP, family planning.

A standard mixed-methods approach, as applied within the EGH programme, was adopted for the implementation of country case studies. This included undertaking quantitative analysis (2022–2023), policy, programme and financing reviews, systematic reviews and qualitative research to answer proposed research questions (table 1). Quantitative trend analysis described historical changes in mCPR, while advanced decomposition techniques identified the specific factors driving these changes. The policy, programme and financing reviews captured long-term shifts in national strategies, service delivery models and funding patterns, including the influence of donor transitions and external shocks, and linked these to observed fertility and FP outcomes. This was complemented by a systematic review synthesising global and country-specific evidence on FP progress drivers. Finally, qualitative interviews with policymakers, providers and community actors filled information gaps, validated quantitative findings and offered nuanced explanations for why and how change occurred (as shown in figure 2). Together, these components created a robust, triangulated evidence base for understanding country pathways to FP progress.

**Table 1:** Mixed methods research questions in the Exemplars in Family Planning case studies

	Research question	Research method
Ecological factors	What is the role of major ecological factors (eg, politics, leadership, international agencies) in influencing the family planning landscape?	Programme, policy and finance review
		Qualitative research
		Systematic review
Individual level factors	Which socioeconomic development and ecological factors were especially impactful in increasing women’s ability to exercise their rights, make their own choices about timing and method of contraception?	Quantitative analysis
		Qualitative research
Healthsystems	What are the drivers of success in terms of demand and supply-side policies and interventions, and what are their relative contributions? Can we establish the sequencing of policy and programmatic interventions (demand and supply) and pathways leading to accelerated change in demand satisfied for family planning and modern contraceptive prevalence?	Programme, policy and finance review
		Quantitative analysis
		Rapid desk review
Community-level factors	How were the rights of women and vulnerable groups (such as adolescent girls and boys, younger couples and those living in remote areas, belonging to a particular religion or ethnic group) addressed?	Qualitative research
		Programme, policy and finance review
		Systematic review

## Quantitative research

For each of the six countries studied, nationally representative data were used to calculate mCPR and demand satisfaction at each available time point between 1986 and 2020 (see table 2 for data sources). Because countries were selected using pre-COVID-19 data, these analyses used only pre-pandemic data to avoid capturing the pandemic’s impacts on FP. Gaps in mCPR were examined over time by education, age, subnational region and parity to identify populations making more progress than others and to probe into these differences in the qualitative component.

**Table 2:** Data sources available and used by selected countries to perform quantitative analysis from 1986–2020

Countries	Data sets used in descriptive analyses	Data sets used in decomposition or risk factor analyses
Kenya	DHS: 1993, 1998, 2003, 2008, 2014 UNPD: 1993–2020 PMA: 2019, 2021	DHS: 2003, 2014
Malawi	DHS: 1992, 2000, 2004, 2010, 2015 UNPD: 1992–2020	DHS: 2004, 2015
Senegal	DHS: 1986, 1992–1993, 1997, 2005, 2010–11, 2012–13, 2014, 2015, 2016, 2017, 2018, 2019 UNPD: 1990-2020	DHS: 2005, 2019
Sierra Leone	DHS: 2008, 2013, 2019 MICS: 2017 UNPD: 1990–2020 UNFP: 2020	DHS: 2008, 2019
Lao People's Democratic Republic	RHS: 1995, 2000, 2005 UNPD: 1990–2020	RHS: 2005 LSIS/MICS: 2012, 2017
Bolivia	DHS: 1989, 1994, 1998, 2003, 2008 UNPD: 1990–2020 UNFP: 2020	DHS: 2003 EDSA: 2016

DHS, Demographic and Heath Survey; EDSA, Encuesta de Demografía y Salud; LSIS, Lao Social Indicator Survey; MICS, Multiple Indicator Cluster Surveys; RHS, Reproductive Health Survey; UNFP, United Nations Family Planning; UNPD, United Nations Population Division.

Drivers of mCPR were identified using the Oaxaca-Blinder decomposition method.^25 26^ The analysis drew on individual-level survey data from the closest available to 2000 and 2020. The sample comprised sexually active women aged 15–49 years, excluding pregnant, menopausal and infecund women. Only respondents with complete data were included and only independent variables with data at baseline and endline were included. Analyses were stratified by age (15–24 years, 25–49 years and all ages) and relationship status (all married and married/in-union women), resulting in six models per country (see online supplemental file 1).

Models were intended to have identical independent variables across countries but varied slightly based on data availability (eg, barriers to accessing healthcare items were not included in the Kenya models because they were not included in the 2003 Kenya Demoghraphic Health Survey). Findings were used to add and modify questions in the qualitative data collection guides and to conduct cross-country comparisons.

## Systematic reviews

Systematic reviews were used to synthesise known socioecological determinants and drivers of improved FP outcomes in the selected countries between 1990 and 2024. The primary outcome of interest was mCPR and secondary outcomes were unmet need and demand satisfied.

Searches were conducted in MEDLINE, EMBASE, APA PsycINFO, CINAHL, Web of Science and JSTOR, using a standardised search strategy (online supplemental file 2). Eligible studies reported primary data, were nationally or subnationally representative, focused on women and men of reproductive age and adolescents (10–19 years), and examined at least one determinant of FP outcomes. Experimental designs, randomised controlled trials, qualitative methods, systematic reviews, protocols and studies focusing exclusively on high-risk or vulnerable populations were excluded. Data were extracted on study characteristics, population demographics, outcomes, analytical methods and relevant programme or policy contexts.

Evidence was analysed across multiple ecological levels, including individual, couple, community, environmental, policy, population and geographic factors. Results were used to inform other components of the case studies. Data on factors associated with FP were incorporated into quantitative analyses and data collection guides (through revisions) to enhance historical and contextual understanding of findings.

## Policy, programme and financing reviews

To gain an indepth, context-specific understanding of policies, strategies and programmes adopted in select countries that may have contributed to improved FP outcomes, each country team mapped relevant policies, programmes, interventions, resource allocation and expenditure commitments from 1990 to 2020. Programmes and policies were identified by reviewing the published and grey literature, Ministry of Health documents, national strategies and programme evaluations. For each entry, information on the type of intervention, year of introduction, objectives, target populations, implementation rationale, budget or expenditure, evaluation findings and status were extracted. The final list was developed and vetted during consultative stakeholder group meetings in each country.

**Figure 3:**
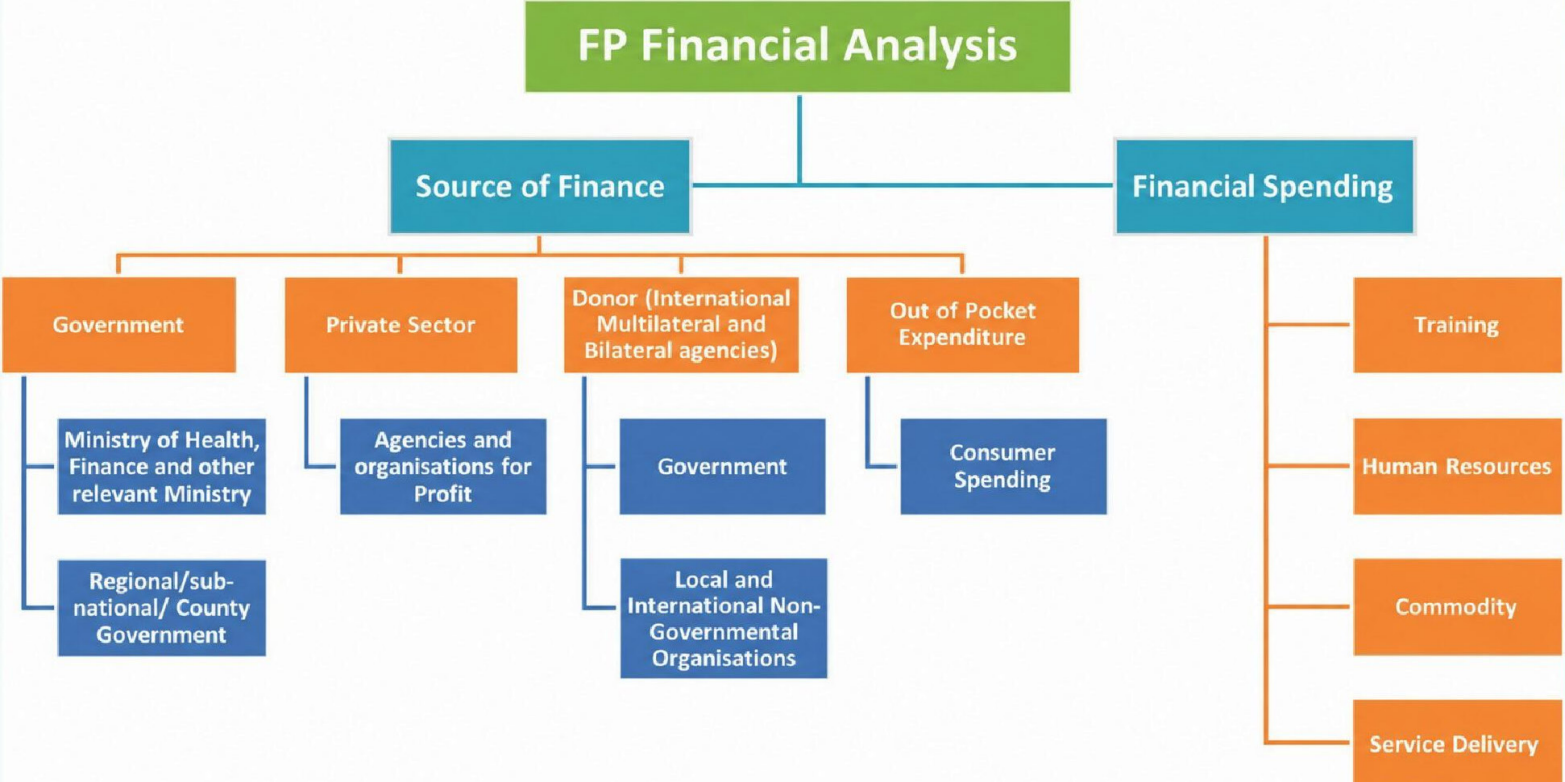
Type of financial data used in the analysis. FP, family planning.

A similar approach was used to analyse financial data, drawing on government budgets, donor reports (from organisations such as World Health Organization, World Bank, United Nations Funds Population Association, United States Agency of International Development, Organization for Economic Co-operation and Development, Gates Foundation), National Health Accounts, costing studies and grey literature reports. Data on FP allocations, expenditures and funding flows were compiled to assess trends in financial commitment, spending patterns and sustainability of investments (figure 3).

A timeline of key policies, programmes and financial commitments was then mapped against annual changes in mCPR and demand satisfaction to identify the most influential policies and programmes shaping FP outcomes. Timelines were used in qualitative interviews to anchor reflections around periods of high and low modern contraceptive use, enabling exploration of how policy, programme and financial changes influenced uptake.

Qualitative research to understand the drivers of changes in FP outcomes was conducted across all six countries. Key informant interviews with national experts, policymakers and programme implementers provided system-level insights on policies, service delivery, financing and contextual enablers. Focus group discussions (FGDs) captured community and user perspectives, exploring lived experiences, sociocultural norms, fertility intentions and barriers to contraceptive uptake and validated whether programme improvements effectively reached diverse populations, including adolescents (online supplemental file 3).

The interviews employed open-ended, semistructured guides (online supplemental file 4), informed by quantitative findings, the systematic review and the Consolidated Framework for Integrated Research^27^ and country-level feedback with questions tailored to each participant group. Using both in-depth interview and focus group discussion approaches enabled data triangulation and yielded rich insights into individual and collective perspectives on contraceptive service experiences and sociocultural influences. Integrating these qualitative findings with quantitative results strengthened interpretation and provided a nuanced understanding of systemic, programme, and behavioural drivers of FP progress. This ensured that changes were not only measured but also meaningfully explained within each country context, identifying key enablers, barriers and implications for policy and programme improvement.

## Data triangulation

We undertook triangulation across systematic reviews, quantitative analyses, qualitative research, policy, programme and financing reviews to strengthen the validity of findings, contextualise trends and identify both country-specific and cross-country patterns. Consortium and country workshops, including a multi-country meeting (held in February 2024), were instrumental in validating results, refining interpretations and confirming convergence and divergence across multiple data sources.

While triangulation strengthened confidence in the results, some limitations remain. High data availability requirements for decomposition analysis limited the inclusion of Lao PDR in the cross-country comparison. Variability in survey years across some datasets (eg, DHS, MICS, EDSA) and gaps in subnational, method-specific and potential driver data also necessitated careful integration with qualitative and policy evidence. Given the sensitive nature of FP, the qualitative data collected from the community are subject to response bias, though country research teams attempted to mitigate this in culturally sensitive ways. Evaluations were unavailable for several policies identified as likely drivers of progress, limiting the extent to which that progress can be attributed to any single policy. Some countries (eg, Lao PDR) had little published research available on drivers of FP progress. These limitations were addressed in the case studies by triangulating findings across methods and through iterative discussions among the entire research team.

## Synthesis

A consultative workshop was held in February 2024, attended by principal investigators from each case study country, TAG and consortium members. Findings and key lessons from case studies including all phase 1 country research activities and phase 2 systematic reviews and quantitative analyses were presented. Guided by the conceptual framework, participants aligned cross-cutting themes and drivers of FP progress. These included strong governance and political commitment, innovative and sustainable financing, health system strengthening, community engagement and demand generation, supportive policy and legal environments and adolescent-focused SRH services. Phase 2 countries used the themes and drivers to guide their remaining research activities.

Each country’s findings were systematically consolidated against the agreed key thematic domains, with country teams aligning their qualitative, quantitative and policy evidence to these core drivers of FP progress. Gaps and specific context insights were identified and further investigated. This structured alignment enabled countries to iteratively build and refine their findings, ensuring that results were both locally grounded and comparable across settings, while contributing to a coherent cross-country understanding of factors influencing progress.

## Conclusion

This work demonstrates how a multicountry analysis supported by a consortium of experienced researchers and the leadership of local researchers can enhance methodological rigour and improve relevance in FP research. By employing a mixed-methods design that engaged both TAG and national stakeholders at the country level, we contextualised findings, facilitated cross-country learning and strengthened the validity of the results.

The synthesis identified cross-cutting drivers of FP progress in FP exemplar countries. Despite some data limitations, the project provides evidence-informed guidance to accelerate modern contraceptive uptake and improve demand satisfaction, while emphasising interventions tailored to local socio-cultural, political and health system contexts. Future research should focus on longitudinal evaluations and broader country coverage to deepen the understanding of sustainable FP progress at a global scale.

## Supplementary material

10.1136/bmjgh-2024-018769 online supplemental file 1

10.1136/bmjgh-2024-018769 online supplemental file 2

10.1136/bmjgh-2024-018769 online supplemental file 3

10.1136/bmjgh-2024-018769 online supplemental file 4

## Data Availability

Data are available upon request.

## References

[1] Joshi R, Khadilkar S, Patel M (2015). Global trends in use of long-acting reversible and permanent methods of contraception: Seeking a balance. Int J Gynaecol Obstet.

[2] Indicator WB (2017). International bank for reconstruction and development.

[3] Cleland J, Conde-Agudelo A, Peterson H (2012). Contraception and health. Lancet.

[4] United Nations Department of Economic and Social Affairs PD. (2022). World Family Planning 2022:. UN DESA/POP/2022/TR/no.4.

[5] United Nations Department of Economic and Social Affairs PD (2020). World fertility and family planning 2020: Highlights.

[6] United nations department of economic and social affairs pd (2022). Population Division (2022) UN DESA/POP/2022/TR/no.3. This report is available in electronic format on the Division’s website at www.

[7] Fund UNP (2018). The power of choice: Reproductive rights and the demographic transition.

[8] Singh S DJ, Ashford LS (2014). Adding It Up: The Costs and Benefits of Investing in Sexual and Reproductive Health New York: Guttmacher Institute.

[9] World Health Organization Department of Sexual and Reproductive Health and Research (WHO/SRH) and Johns Hopkins Bloomberg School of Public Health/ Center for Communication Programs (CCP) (2022). Knowledge SUCCESS. Family Planning: A Global Handbook for Providers (2022 Update).

[10] Tignor M, Geddes CE, Dilaverakis Fernandez A (2025). Adding It Up 2024: Investing in Sexual and Reproductive Health in Low- and Middle-Income Countries.

[11] Souza JP, Day LT, Rezende-Gomes AC (2024). A global analysis of the determinants of maternal health and transitions in maternal mortality. Lancet Glob Health.

[12] Pradhan MR, Mondal S (2023). Examining the influence of Mother-in-law on family planning use in South Asia: insights from Bangladesh, India, Nepal, and Pakistan. BMC Womens Health.

[13] Memon ZA, Fazal SA (2024). Effective strategies for increasing the uptake of modern methods of family planning in South Asia: a systematic review and meta-analysis. BMC Womens Health.

[14] Mutumba M (2022). Mass media influences on family planning knowledge, attitudes and method choice among sexually active men in sub-Saharan Africa. PLoS One.

[15] Najmi H, Ahmed H, Halepota GM (2018). Community-based integrated approach to changing women’s family planning behaviour in Pakistan, 2014-2016. Public Health Action.

[16] Sharan M VT (2002). Spousal communication and family planning adoption: effects of a radio drama serial in Nepal. Int Fam Plan Perspect.

[17] Fagan T, Dutta A, Rosen J (2017). Family Planning in the Context of Latin America’s Universal Health Coverage Agenda. Glob Health Sci Pract.

[18] Tsui AO, McDonald-Mosley R, Burke AE (2010). Family planning and the burden of unintended pregnancies. Epidemiol Rev.

[19] Cardona C, Rusatira JC, Salmeron C (2025). Progress in reducing socioeconomic inequalities in the use of modern contraceptives in 48 focus countries as part of the FP2030 initiative between 1990 and 2020: a population-based analysis. Lancet Glob Health.

[20] Memon ZA, Mian A, Martopullo I (2026). Family planning exemplar country selection methodology: time lag and trends analysis. BMJ Glob Health.

[21] Fund. UNCs (1990). Conceptual Framework on Maternal and Child Nutrition. Strategy for Improved Nutrition of Children and Women in Developing Countries.

[22] Enyinnaya JC, Anderson AA, Kelp NC (2024). The Social Ecology of Health Beliefs and Misinformation Framework: Examining the impact of misinformation on vaccine uptake through individual and sociological factors. Vaccine (Auckl).

[23] AS. A (2024). Behavior Change Theories and Models Within Health Belief Model Research: A Five-Decade Holistic Bibliometric Analysis. Cureus June.

[24] Edmeades J, Mejia C, Parsons J (2018). (A conceptual framework for reproductive empowerment: Empowering individuals and couples to improve their health (background paper).

[25] Oaxaca R (1973). Male-Female Wage Differentials in Urban Labor Markets. Int Econ Rev (Philadelphia).

[26] Słoczyński T (2020). Average Gaps and Oaxaca–Blinder Decompositions: A Cautionary Tale about Regression Estimates of Racial Differences in Labor Market Outcomes. *ILR Review*.

[27] Damschroder LJ, Aron DC, Keith RE (2009). Fostering implementation of health services research findings into practice: a consolidated framework for advancing implementation science. Implementation Sci.

