# Supplementary material for: Assessing family planning progress in exemplar countries: development of a conceptual framework and case study methodology
Source: BMJ Glob Health. 2026 Jun 9;11(Suppl 3):e018769. doi: 10.1136/bmjgh-2024-018769 (PMC13250193; doi:10.1136/bmjgh-2024-018769)
Supplement: online supplemental file 1 [file bmjgh-11-Suppl_3-s001.docx]

# **Decomposition Model**

| **Variable Availability Oaxaca-Blinder Decomposition Analysis Evaluation Sample in Senegal (SN), Malawi (MW), Kenya (KE), Sierra Leone (SL), and Bolivia (BO)** | | | | | | | | |
| --- | --- | --- | --- | --- | --- | --- | --- | --- |
|  | | | | **Country** | | | | |
| **Analysis** | **Variable** | **Definition** | **Level of measurement** | **SN** | **MW** | **KE** | **SL** | **BO** |
| All and married/in-union women analyses | Education | Years of education | Individual | Y | Y | Y | Y | Y |
|  | Employed in the last 12 months | 1 = respondent has worked in the past 12 months, is currently working, or has a job but has been on leave for the last 7 days; 0 = respondent has not worked in the past 12 months or missing | Individual | Y | Y | Y | Y | N |
|  | Relationship status | 1 = married or living with a partner as if married; 0 = never married, divorced, separated, or widowed | Individual | Y | Y | Y | Y | Y |
|  | Age at first sex | Mean age | Individual | Y | Y | Y | Y | Y |
|  | Urban residence | 1 = urban, 0=rural | Individual | Y | Y | Y | Y | Y |
|  | Wealth index | Computed at SickKids using principal components analysis (range: 1-10) | Individual | Y | Y | Y | Y | Y |
|  | Attitudes towards wife-beating | Calculated using responses on 5 survey items (depending on availability) about whether the respondent feels it is justified for a man to hit his wife in the following scenarios: | Individual | Y | Y | Y | Y | Y* |
|  |  | (1) she goes out without telling him |  |  |  |  |  |  |
|  |  | (2) she neglects the children |  |  |  |  |  |  |
|  |  | (3) she argues with him |  |  |  |  |  |  |
|  |  | (4) she refuses to have sex with him |  |  |  |  |  |  |
|  |  | (5) she burns the food |  |  |  |  |  |  |
|  |  | Responses were recoded as justified = 1, don’t know = 0; **not** justified = -1 and a score was generated by adding the responses. Range: -5 to +5 depending on data availability. A high score indicates more agreement that wife-beating is justified, and a low score indicates less agreement that wife-beating is justified.  *BO: Married/in-union respondents only; considered at ecological level for all women analysis |  |  |  |  |  |  |
|  | Decision-maker on respondent's healthcare | Responses were recoded as husband/partner alone or other alone makes decision = 0, and respondent alone makes decision or joint decision with partner/other person = 1. A high score indicates more decision-making power from respondent and a low score indicates less decision-making power from respondent. Entered at ecological level for all women and individual level for married/in union analysis | Ecological | Y | N | N | Y | Y |
|  | Decision-maker on contraceptive use | Responses were recoded as husband/partner alone or other alone makes decision = 0, and respondent alone makes decision or joint decision with partner/other person = 1. A high score indicates more decision-making power from respondent and a low score indicates less decision-making power from respondent. | Ecological | N | N | N | N | Y |
|  |  |  |  |  |  |  |  |  |
|  | Decision-making power | Calculated using responses on 2 or 3 survey items (depending on availability) about who usually decides on decisions in the following scenarios (entered at ecological level for all women and individual level for married/in union analysis ):  (1) large household purchases; (2) visits to family or relatives; (3) what to do with money husband earns; (4) food to be cooked.  Responses were recoded as husband/partner alone or other alone makes decision = -1, and respondent alone makes decision or joint decision with partner/other person = 1, and a score was calculated summing all the responses. A high score indicates more decision-making power from respondent and a low score indicates less decision-making power from respondent.  *SN had large household purchases and visits to family or relatives (range -2 to +2)  *MW had large household purchases and visits to family or relatives (range -2 to +2)  *KE had large household purchases, visits to family or relatives, and food to be cooked each day (range -3 to +3)  *SL had large household purchases, visits to family or relatives, and what to do with money husband earns (range -3 to +3)  *BL had large household purchases and visits to family or relatives (range -2 to +2) | Ecological | Y * | N | N | Y | Y |
|  | Distance to facility | Distance to facility is a big problem when seeking medical advice/treatment: 1=yes; 0=no | Individual | Y | Y | N | Y | Y |
|  | Money | Getting money is a big problem in seeking medical advice/treatment for self: 1=yes; 0=no | Individual | Y | Y | N | Y | Y |
|  | Permission | Getting permission to go is a big problem in seeking medical advice/treatment for self: 1=yes; 0=no | Individual | Y | Y | N | Y | Y |
|  | Not wanting to go alone | Not wanting to go alone is a big problem in seeking medical advice/treatment for self: 1=yes; 0=no | Individual | Y | Y | N | Y | Y |
|  | Recent exposure to FP messages on radio | 1 = yes, had recent exposure (i.e., last few months); 0 = no recent exposure | Individual | Y | Y | N | Y | Y |
|  | Recent exposures to FP messages on TV | 1 = yes, had recent exposure (i.e., last few months); 0 = no recent exposure | Individual | Y | Y | N | Y | Y |
|  | Recent exposure to FP messages through newspapers/ magazines | 1 = yes, had recent exposure (i.e., last few months); 0 = no recent exposure | Individual | Y | Y | N | Y | Y |
|  | Source of FP supplies | 1 = Respondent’s source of family planning supplies was from a private clinic or pharmacy; 0 = Respondent’s source of family planning supplies was from a government clinic/pharmacy or government home/community delivery | Ecological | N | N | N | N | N |
|  | Recently visited by FP fieldworker | 1 = yes, visited by family planning fieldworker in the past 12 months; 0=not recently visited | Individual | Y | Y | Y | Y | N |
|  | Recently visited health facility | 1 = yes, visited health facility in the last 12 months; 0=no recent visits | Individual | Y | Y | N | Y | N |
|  | Total number of modern methods ever heard of (range: 0-11) | A sum of the number of modern family planning methods a respondent has ever heard of out of 11:  (1) female sterilization; (2) male sterilization; (3) contraceptive pill; (4) IUD; (5) injectables; (6) implants; (7) male condom; (8) female condom; (9) lactational amenorrhea method (LAM); (10) standard days method (SDM); (11) emergency contraception  *SN excludes SDM (n/a in 2005)  *BL excludes SDM (n/a in either year) and includes contraceptive foam/jelly  *ML excludes SDM & LAM  *KE excludes SDM & LAM (n/a in 2003)  *SL excludes SDM (n/a in 2008) | Individual | Y* | Y* | Y* | Y* | Y* |
|  | Child loss | 1=any sons or daughters who have died or ever had a terminated pregnancy; 0=no sons or daughters have died or never had a terminated pregnancy | Individual | Y | Y | Y | Y | Y |
|  |  |  |  | | | | | |
| Married/in-union women analyses only | Age at first cohabitation | Age in years at first cohabitation with partner | Individual | Y | Y | Y | Y | N |
|  | Age difference between respondent and partner | Respondent’s age in years subtracted by partner’s age in years | Individual | Y | Y | Y | Y | Y |
|  | Education difference between respondent and partner | Respondent’s education in years subtracted by partner’s education in years | Individual | Y | Y | Y | Y | N |
|  | Decision-maker on respondent's healthcare | Responses were recoded as husband/partner alone or other alone makes decision = 0, and respondent alone makes decision or joint decision with partner/other person = 1. A high score indicates more decision-making power from respondent and a low score indicates less decision-making power from respondent. | Individual | Y | Y | Y | Y | Y |
|  | Decision-making power | Calculated using responses on 2 or 3 survey items (depending on availability) about who usually decides on decisions in the following scenarios: (1) large household purchases; (2) visits to family or relatives; (3) what to do with money husband earns; (4) food to be cooked.  Responses were recoded as husband/partner alone or other alone makes decision = -1, and respondent alone makes decision or joint decision with partner/other person = 1, and a score was calculated summing all the responses. A high score indicates more decision-making power from respondent and a low score indicates less decision-making power from respondent.  *SN had large household purchases and visits to family or relatives (range -2 to +2)  *MW had large household purchases and visits to family or relatives (range -2 to +2)  *KE had large household purchases, visits to family or relatives, and food to be cooked each day (range -3 to +3)  *SL had large household purchases and visits to family or relatives (range -2 to +2)  *BO had large household purchases and visits to family or relatives (range -2 to +2) | Individual | Y* | Y* | Y* | Y* | Y* |
|  | Decision-maker on contraceptive use | Responses were recoded as husband/partner alone or other alone makes decision = 0, and respondent alone makes decision or joint decision with partner/other person = 1. A high score indicates more decision-making power from respondent and a low score indicates less decision-making power from respondent. | Ecological | N | N | N | N | Y |
