# Supplementary material for: Assessing family planning progress in exemplar countries: development of a conceptual framework and case study methodology
Source: BMJ Glob Health. 2026 Jun 9;11(Suppl 3):e018769. doi: 10.1136/bmjgh-2024-018769 (PMC13250193; doi:10.1136/bmjgh-2024-018769)
Supplement: online supplemental file 3 [file bmjgh-11-Suppl_3-s003.docx]

| Description of Potential Participants in FDGs and IDIs | | |
| --- | --- | --- |
| **Participant Category** | **Characteristics** | **Exclusion Criteria** |
| Healthcare workers | Individuals with experience in delivering family planning and reproductive health services in the community. | Healthcare workers professionals who specifically lack work experience in family planning and reproductive health. |
| Community influencers | Individuals who influence decision making around family planning and reproductive health services. | Individuals with personal or professional conflicts of interest related to family planning or specific methods.  Individuals residing within communities for less than 5 years. |
| Community beneficiaries  Couples and individual married men and women aged 15-49 | Men and women who have witnessed change in family planning uptake within the community and share a key role in decision making around family planning/sexual and reproductive health (FP/SRH). | Men and women with limited language proficiency. Also, men and women residing within the community for less than 5 years. Adolescents have been residing within the community for less than 5 years. |
| Adolescent boys and girls aged 15-19 years | Adolescent girl and boys were purposively selected to share their understanding, perspectives and experiences on FP/SRH. | Adolescents whose parents/guardians do not provide assent for participation |
| Key informant participants | - National and reginal level program evaluators and personnel from ministry and planning. Interviews with former and current representatives from USAID, UNFPA, MSI, Path finder. - At regional level representative of program implementers and policy makers 3. Senior member of ministry of health, representative from provide organization working in Senegal were targeted for both policy, program and financial level information - Regional and national family planning experts’ family planning professionals, community members and representatives from adolescent’s group.. | Key informants will include leaders with 10 to 20 years of experience in the field, selected for their expertise in policy, programmatic, and financial aspects of family planning and reproductive health services within the selected countries |
