# Supplementary material for: Assessing family planning progress in exemplar countries: development of a conceptual framework and case study methodology
Source: BMJ Glob Health. 2026 Jun 9;11(Suppl 3):e018769. doi: 10.1136/bmjgh-2024-018769 (PMC13250193; doi:10.1136/bmjgh-2024-018769)
Supplement: online supplemental file 4 [file bmjgh-11-Suppl_3-s004.docx]

## **Focus Group Discussion Guide for Young Adolescent Boys and Girls (Age 15-19 years)**

Participant information sheet

Region: ____________________________________________

Date: ___________________________________________

Time of the beginning of the FGD: ______________________________

**List of names and information of participants**

| **Instruction: This sheet should be filled before starting the FGD** | | | | | | | |
| --- | --- | --- | --- | --- | --- | --- | --- |
| **Date:** | | **Town/District:** | | | | **Village:** | |
| **No.** | **Name** | **Age** | **Gender** | **Education** | **Marital Status** | **Occupation/ Profession** | **Respondent Signature** |
| 1 |  |  |  |  |  |  |  |
| 2 |  |  |  |  |  |  |  |
| 3 |  |  |  |  |  |  |  |
| 4 |  |  |  |  |  |  |  |
| 5 |  |  |  |  |  |  |  |
| 6 |  |  |  |  |  |  |  |
| 7 |  |  |  |  |  |  |  |
| 8 |  |  |  |  |  |  |  |
| 9 |  |  |  |  |  |  |  |
| 10 |  |  |  |  |  |  |  |

**General introductory questions**

*I would like to inquire about your daily routine and activities while living in the community.*

1. How do you spend your typical day? What are the activities you do in your free time?

**Probes:** School, Stitching Sewing, Gaming/Sports, Labor, Household chores

1. Do you think these activities are different for boys and girls?

Does anyone from your family support you in such activities? If yes, how and if not why? (Use journey map to record the whole day activities)

1. Can you describe how young boys and girls are educated in your community?
   **Probes:**
   - 1. Are there any difference between girls’ and boys’ education? If yes, what are these differences?

**Knowledge/ awareness and perception on events of health from childhood to adulthood**

*Now I would like to discuss a few SRH related issues such as early marriage, puberty changes, gender base violence, mental physical health, understanding of gender-based issues.*

*For moderator: Please explain each topic and explore the participant perspective in terms of routine practice and need of information and services in community/region.*

1. What do you know about the changes in your body when you are growing up?
   **Probes:**
2. What types of changes occur when girls grow into a woman and boys into a man?
3. Do these changes affect girls’/boys’ physical, mental, and emotional health? If yes, how?
   1. Do you feel comfortable sharing issues such as puberty and other issues with friends, siblings, family, or teacher? Why?
4. What do you believe is the appropriate age for marriage and what are your own preferences in this regard, if any?

**Probes:**

1. What do you think of girls and boys getting married before 18 years old (their health, education, jobs, life aspirations)?
2. Role of parents, relatives in deciding marriage

*For the following questions, ICRPs should explore all possible reasons, potential factors and major interventions implemented to mitigate the risk of early pregnancy in the region or community, particularly in the case of Bolivia, where teenage pregnancy is frequently reported.*

1. Can you describe what you know about early /adolescent pregnancy? Is it wide-spread in your community?
   1. What are your personal beliefs regarding how early /adolescent pregnancy affects girls and boys?

**Probes:**

1. Risks, benefits
2. Social implications

Have you heard of sexually transmitted diseases? If yes, can you describe what you know about them? What is the trend of these infections in your community/region?

**Probes:**

- 1. Disease or symptoms, means of transmission, prevention.
  2. HIV or AIDS
  3. Other infections /disease – please explain.

**Gender Equality and Empowerment**

1. Do you feel that men and women are equal in society? What does the term gender inequality mean to you?

**Probes:**

1. Sexual and reproductive health and rights
2. Equal economic empowerment opportunities
3. Disparities in male and female access to education
4. In your opinion, what does empowerment mean for males and females?
   **Probes:**
5. Decision-making power
6. Ability to influence social change
7. Access to opportunities for empowerment

**Community Support and Service Availability**

*This section will consist of few questions related to clinical services and support through other life skills based services including comprehensive sexuality education in your community/country* ***(specific to last 20 years)***

*For moderator: please emphasize on each SRH area with respect of availability of services and information trend in last 20 years)*

1. Is there any institute/place/person where you received information/guidance on puberty, hormonal/body changes during menstrual period, marriage, contraceptives, pregnancy? If yes, can you please elaborate on them?

**Probes**:

1. Rank sources of information by importance- *(high possibility of information source and service availability)*
2. Role of health care providers in supporting adolescents with this information
   1. How important, do you think, it is to provide SRH knowledge to young boys and girls? And what are various ways through which to provide this knowledge?
      **Probes:**
       a. School curriculum
       b. Community platforms and programs
3. When was the last time you went to see a doctor? What types of healthcare services are available for youth in your community?

**Probes:**

1. Healthcare facilities responsiveness to physical and mental healthcare needs of adolescent boys and girls
2. Accessibility of health facilities for adolescents
3. Role of adolescent-based clinical facilities in addressing adolescent needs

8.1 In your opinion, to what extent is healthcare staff aware of your health needs and preferences?

1. Have you heard of any organization or community group in your area/society who offers youth centered services? Are these newly introduced services or have they existed for long?

**Probes:**

- 1. What kind of information did you receive from that place? Did you receive information on SRH issues among adolescent girls and boys on the following areas:
     1. Early marriage
     2. Sexual/physical violence
     3. Gender in equality,
     4. Unintended pregnancy
     5. Abortion services,
     6. Maternal health
     7. Family planning services
  2. What do you think about these services? Do you think these are helpful for addressing common myths and misconceptions? If yes, please explain how they are helpful?
  3. Are there any kind of services available in your community- providing the same sort of services?

1. Have you heard of comprehensive sexual and reproductive health education (CSE)?  If yes, please describe what you know about it.

**Probes:**

1. What do you know about sexual health education?
2. In your community/district/country have you received sexual and reproductive health education?
3. Does it help equipping young people with the skills to make responsible decisions for their health and well-being?

10.1 Do you think CSE helps advance gender equality and the sexual and reproductive health rights of young people?

**Probes:**

1. How does it affect risky behaviors of young boys/girls including substance abuse
2. Does it address gender and power issues and delay sexual debut

10.2 Is there any course or curriculum on CSE where you or your peers reside?

**Probes:**

- 1. What age group is catered to by these courses?
  2. Is it only for boys or girls?
  3. Is there a fee for these courses?
  4. Do you think there are important learnings for you and your peers from these courses? If yes, elaborate on these learnings

**Family Planning**

*Now, we will discuss the availability of family planning services and information in your community and region.*

1. Have you heard of family planning? How would you define family planning in your own words?

**Probes:**

1. Are there any benefits or harms of using FP methods or services?
2. Are you aware of any family planning services or programs available to adolescents in your community?
3. Who is providing these services? In the public or private sector?
4. Have you ever been counselled on FP methods? Do you use or have you ever used any method?

Probes:

1. If yes, what method have you used? What encourages you to use the method?
2. If not, why not?
3. Do you think contraceptives are easily available/accessible in your community?
4. In your opinion, what are the major barriers or challenges for adolescents your age in accessing family planning services?
   **Probes:**
5. Unavailability of FP services for young people
6. Unavailability of skilled/trainer providers
7. Any other reason/barrier
8. Who decides to use the contraceptive methods among couples?
   1. Male partner usually decides
   2. Other family member decides about the FP use.
   3. It’s mainly women decision
   4. It’s a mutual decision
9. How do you think your community’s culture (general beliefs, values, assumptions) affects FP uptake amongst adolescents?
   **Probes:**
10. Are there misconceptions or myths about family planning that you've come across?
11. Do you feel comfortable discussing family planning topics with your family members or friends? Why or why not?
12. Are there any specific challenges faced by certain groups within the adolescent population in accessing family planning?

**Probes:**

1. How do you define these certain groups – marginalized, special needs
2. Describe, if any, the additional barriers faced by the groups you described
3. Are healthcare providers aware of and responsive to the FP and reproductive health needs of these certain groups
4. Is there anything else you would like to add or share about adolescents, family planning or sexual and reproductive health, that we did not discuss today?

**Conclusion**

Thank you for your time. This was the last question, if you have any thoughts, information, memories that you consider important, please do share!

*Time of the end of FGD*

*________________________*

## **Focus Group Discussion Guide for men and women (Age 15-49 years)**

Region: ____________________________________________

Date: ___________________________________________

Time of the beginning of the FGD: ______________________________

*It is important to emphasize that this discussion is primarily related to participant’s own beliefs and knowledge of family planning programs and use.*

**List of names and information of participants**

| **Instruction: This sheet should be filled before starting the FGD** | | | | | | | |
| --- | --- | --- | --- | --- | --- | --- | --- |
| **Date:** | | **Town/District:** | | | | **Village:** | |
| **No.** | **Name** | **Age** | **Gender** | **Education** | **Marital Status** | **Occupation/ Profession** | **Respondent Signature** |
| 1 |  |  |  |  |  |  |  |
| 2 |  |  |  |  |  |  |  |
| 3 |  |  |  |  |  |  |  |
| 4 |  |  |  |  |  |  |  |
| 5 |  |  |  |  |  |  |  |
| 6 |  |  |  |  |  |  |  |
| 7 |  |  |  |  |  |  |  |
| 8 |  |  |  |  |  |  |  |
| 9 |  |  |  |  |  |  |  |
| 10 |  |  |  |  |  |  |  |

**General introductory questions**

*Introduce this section by saying: “Please remember that we are only talking about your own beliefs and knowledge of family planning programs and use.”*

1. What is your general perception of opportunities for adolescent girls/boys/men and women in your community?

**Probes:**

- 1. Are there equal opportunities for men and women to attain education (also higher education)?
  2. Are there equal job opportunities for men and women in your community?
  3. Can men and women equally exercise their right to marriage, timing of marriage, choice of their partner, child aspirations, family size, sex composition?

**Community based Programs and Intervention**

*I would like to ask you some questions to understand the community’s perspective, acceptability and needs related to the programs and interventions implemented in your region/community.*

1. Do you know of any FP/SRH programs implemented in your community over the last 20 years? If yes, can you please elaborate on them?

**Probes:**

- 1. Do you think there is a need for FP in your community? Is there any program/intervention/strategy implemented in your community that helped or not in terms of meeting the needs of women and girls in your community?
  2. What kind of programs/interventions have been implemented? Can you describe the program? (Voucher initiatives, Outreach Initiatives, Maternal child health and women focused programs, community-based FP awareness programs, FP commodity supply initiatives, etc.)
  3. Describe what differences you have observed in terms of awareness and usage of FP methods/services over the last 20 years ?
- ***Awareness:***
- *How does the community awareness raising campaign work in your community/region? Is it through community health workers, social media (TV, Radio and other digital source involved?*
- *Would you able to describe the role of health care providers (government/provider) to other health care staff in raising the FP awareness in your region/community?*
- ***Method mix trend****: (please use the country quant results to probe on preferences related to different methods)*

1. To what extent were your needs and preferences considered when implementing these FP programs?
2. What is the current trend in the use of modern contraceptives? Which family planning methods do women prefer to use and why?

- ***SierraLeone:*** Implant, Injectables, and Pills are high in use.
- ***Lao PDR:*** Pills and Injectables are high in demand.
- ***Bolivia:*** Female sterilization, pills and IUDs are more common in use.

1. Are there any natural/traditional methods that women used in your community/region?

**Probes:**

1. What is the most common natural method that men and women are using in your community/region?
2. Is there any program/intervention- focused on the use of natural methods in the last 20 years?
3. Why do you think women prefer using natural/traditional family planning methods?
4. Are media and healthcare workers/providers playing a role in encouraging the use of traditional methods?
5. Has the program provided any benefits to you or your community, such as changes in behavior, improved access to and clarity of information related to family planning/sexual and reproductive health services, etc.?

**Probes:**

- 1. Programs/intervention focusing on husband/men involvement in family planning
  2. Programs/initiatives focusing on Community-Based Distribution
  3. Program/interventions focusing on service integration (inclusion of family planning service along with immunization, maternal and child health care services, post pregnancy services, school level sexual health programs for young girls. etc)
  4. Community mobilization – community events/support groups to promote dialogues and encourage the use of family planning.
  5. Interpersonal communication- one to one couple counseling strategy to promote the use of family planning.
  6. Any other intervention/strategy for behavior change in the community, please explain.

*(Note for moderator: It is important to explore each concept mentioned here to understand any linkages to FP use and behavioral change)*

**Social Cultural Context of Family Planning use in the country**

*Now I have some questions to understand the social cultural context of family planning in your region/community over the last 20 years*

1. What cultural and/or local factors do you feel affect your usage of family planning methods? Have these factors changed over the last 15-20 years?

**Probes:**

1. Your ability to make decisions.
2. Decision for the timing of the first birth
3. Preference for a male child
4. Family size
5. Freedom to express sexuality
6. Personal beliefs, your communication with your partner
7. Maintain an equal right and control over economic resources
8. Decision for yourself and family
9. Practice equal access to resources.

***(Note: It is important to explore the linkages of each concept on FP information and services.)***

1. Have negative and positive influences in your social circle, lack of support from husband and family, cultural beliefs and norms, myths and misconceptions about FP methods and side-effects or mass media campaigns influenced your usage of FP methods?
2. Do you think the factors listed below influence the use of contraceptives? If yes, how?
   - 1. Women belong to rich and poor community
     2. Communities dependent on labor migration
     3. Community living in urban vs community living in rural
     4. Availability of family planning services and delivery source (community health worker, private provider, government clinics, any outreach services etc.?
     5. Individual financials (Affordability of services)
     6. Role of education (women with basic education and women with higher education
     7. Women with number of children (0-2, 3-4, 4-5 and 5 plus)
     8. Age of women (young women 15-19 years – mature/adult group 19-30, 31-40 and 40 plus
     9. Role of religion
     10. Quality of services (e.g., poor management of side effects, unwelcoming provider attitudes, insufficient staff for client load, incomplete information on available FP methods and services)
     11. Lack of knowledge about FP methods and services
     12. Sociocultural/religious issues (e.g., myths/misconceptions, biases, sex of staff)
     13. Additional country specific probes to be added by country team.

**Accessibility of Family planning Services**

*Now I would like to ask questions related to the accessibility of family planning services in your community/region over the last 20 years.*

1. Has the availability or access to FP/SRH commodities/information and services changed over time in your village/region?

**Probes:**

1. Can you describe these changes?
2. Participation in the community engagement groups/support groups
3. Establishment of outreach facilities
4. Improvement youth friendly services, life skills-based education (LSBE) resources
5. Availability of commodities
6. What do you think caused these changes?
7. Have you always had access to all contraceptive methods /or the contraceptive method of your choice?
8. Do you believe the availability of certain FP contraceptives has changed over time?
9. Did you previously consider accessing FP information and use to be of importance in your life?
10. If modern contraceptive methods are inaccessible, is there any other method women/men use?
11. Additional country specific probes to be added by country team.

**Quality of Family planning Services**

*Let’s discuss about quality of family planning services at clinical level and community level.*

1. Has your ability to access care at healthcare facilities changed over time in your community? Can you describe what has changed?

**Probes:**

- 1. Has the number of health workers/working hours and clinic distance changed over time?
  2. What about the services provided? (e.g., types, quality, cost, exposure to method related side effects)
  3. Do you observe any discrimination based on gender regarding FP/SRH services and facilities?

1. In terms of equity, do you believe you have equal access to FP/SRH rights and services, regardless of your age, marital status, education, social stigma, socio-economic status, gender, ethnicity, or residence?

- **Bolivia:** disparities in family planning services was observed in region wise and age-related disparities were also observed women living in southern and northern region.
- **Seirra Leone:** Total Fertility Rate is highest in rural areas where most of the population lives. Moreover, outbreaks affected service delivery in some parts of the country – please explain the situation in your region.
- **Lao PDR:** Greater distance from health facilities associated to low use of family planning services similarly, FP services are not available for young people in few regions in the country.

**Challenges related to family planning services.**

1. In your opinion, what are the biggest constraints that you and your community face in accessing FP?

**Probes:**

1. Accessibility (e.g., geographical location, facility hours, waiting times, gender related services)
2. Financial barriers (e.g., fees for methods, supplies, or consultation)
3. Quality of services (e.g., poor management of side effects, unwelcoming provider attitudes, insufficient staff for client load, incomplete information on available FP methods and services)
4. Lack of knowledge about FP methods and services
5. Method related side effects and other health complications
6. Sociocultural/religious issues (e.g., myths/misconceptions, biases, sex of staff)
7. Additional country specific probes to be added by country team

***Conclusion***

1. As we near the end of our interview, is there anything you would like to add to our discussion today?
2. We spoke about programs and interventions implemented in your region/community and cultural and societal norms related to acceptability of family planning . Is there anything else you would like to add that we didn’t discuss today?

*Time of the end of FGD*

## **In-Depth Interview Guide for Key Informants**

##

Thank you for participating in this interview. The aim of this project is to conduct an in-depth assessment of the determinants, specifically political commitment, government policies, population policies, and FP/SRH programs at national and sub-national levels, that may have impacted family planning outcomes in the country from 2000-2020.

Types of key informants related to FP/SRH that may be interviewed include:

- - 1. Healthcare Providers
    2. Community Influencers
    3. Provincial district/village
    4. program managers (e.g., Director General of Health at provincial level),

Name________________________________________________________________________

Village/ ____________________________________________________________

Occupation__________________________________________________________________

Organization________________________________________________________________

Age__________________________________________________

Gender________________

Contact information________________________________________________________

Date of interview___________________________________________________________

**Background and work experience**

1) Briefly tell us about your role in relation to Family Planning / SRHR. Which aspects of FP provision do you have experience with?

1. Service provision, demand generation, policy development, advocacy, research / monitoring / evaluation, etc.
2. Are there specific policies / programs related to FP that you have been involved in drafting, implementing, or funding? Which ones? What was your role?
3. Are there specific political commitments, policy endorsements or legislation that serve as guiding frameworks for your work? If so, which ones?
4. How are FP services delivered in your country? Is FP a separate vertical program or is it integrated into other parts of the healthcare system (e.g., primary care)?

**Family planning trends and drivers**

We would like to ask you about trends in a few family planning metrics in your country between 2000 and 2020.

2) For each trend, please explain what you feel are the main drivers of the patterns observed. Trends: a) mCPR, b) demand satisfied, c) equity gaps based on wealth, education, urban/rural residence, age  
**Probes:**

1. Any explanation for any prominent spikes or dips in the metrics
2. Formal changes like policymaking, law-making, creation of programs and organizational changes
3. Informal changes like shifts in cultural and societal norms, social advocacy efforts, political events
4. Health system changes, changes to health access, increase in health integration models, health care decision-making
5. Policies or programs not explicitly focused on family planning that may have impacted family planning use?
   - 1. In your opinion, have there been collaborative efforts between health and non-health departments (e.g., social welfare, education, etc.) in implementing FP initiatives? 
        **Probes:**
6. What was the role of other health initiatives (e.g., HIV, UHC, MNCH) in improving FP uptake?

**Accessibility**

- - 1. Has the accessibility and availability of FP/SRH health services changed over time in your country/region?

1. If not already discussed in question 6, probe:
2. What has changed (number of health facilities, distance to health facilities, number of health workers (paid or volunteer), number of health workers providing FP information/services, etc.)?
3. What do you think caused these changes? (Inclusion of voucher programs, outreach services, social marketing networks, provider incentive programs, media campaigns, Community based FP education sessions, commodity security)
4. What has changed in terms of providing FP/SRH information to the community (digital technology, door to door counselling, community-based distribution, education, access to information)?
5. Have you also observed the trend of family planning methods between traditional methods to modern method?

- What are the reasons of this change or shift?
- What methods are more commonly used? (From modern methods and from traditional methods)
- Is there any issue with method related side effects management and other health complications?

1. Do you think FP/SRH service provision to vulnerable groups and adolescents has changed over the last 20 years? (Information on SRH services, creating adolescent friendly services, and are they exclusive of gender discrimination)
2. How widespread would you say these changes are?
3. Has there been any change regarding financial barriers to FP/SRH services?
4. Have these changes translated to changes in health seeking behavior/service uptake?

**Role of Public and Private sector**

- - 1. Has the private sector played a significant role in family planning service delivery and accessibility?  
       **Probes:**

1. Service delivery points including drug stores, pharmacies, social marketing, and franchising
   - 1. Describe the role of public sector and delivery points?
     2. Have there been any public-private partnerships? How have they affected FP service delivery, outreach, and coverage? 
        Probes:
        1. Impact on remote / marginalized communities

**Demand**

- - 1. What cultural and/or local factors do you feel impact demand for family planning? Have these factors changed over the last 15-20 years?

**Probes:**

- - - 1. Cultural beliefs and norms, women’s empowerment, agency, partner’s education/knowledge of FP
    1. Have there been any interventions aimed at influencing these factors (e.g., mass media campaigns, social media, social / behavior change initiatives, etc.)? Have they been successful, and if so, why?

**Remaining challenges and recommendations**

- - 1. What factors have impeded progress in improving family planning over the last 20 years, at the national and regional levels?
    2. What do you think are the main challenges for implementing FP/SRH programs at national/sub national/regional level?

**Probes:**

- - - 1. Cost, lack of resources, lack of skilled human resources, donors’ interests, indicators, political commitment, social cultural barriers etc.
    1. In your opinion, what are the biggest constraints that members of the community face in accessing FP/SRH?

**Probes:**

- - - 1. Accessibility /time barriers (e.g., facility hours, waiting times)
      2. Financial barriers (e.g., fees for methods, supplies, or consultation)
      3. Quality of services (e.g., poor management of side effects, unwelcoming provider attitudes, insufficient staff for client load)
      4. Lack of knowledge of the benefits of FP, Sociocultural issues (e.g., myths/misconceptions, biases, sex of staff, agency and FP Decision making, control of economic resources ideal family size, spouse communication, dominance of a certain gender myths and Misconceptions related to FP)
      5. Additional country specific probes to be added by in- country team
    1. What recommendations would you make to policymakers and program managers in other countries who are looking to make progress in family planning?

**Conclusion**

- - 1. As we near the end of our interview, is there anything you would like to add to our discussion today?

*Time of the end IDI*

*________________________*

**Consent form**

**Title of Research Project**:

Exemplars in Family Planning

**Introduction**
Thank you for participating in this interview. This interview is part of the Exemplars in FP project which aims to study countries that have achieved exemplary performance in family planning outcomes. Through this interview, we would like to understand strategies adopted by your country that have contributed to this success and hope to gain an in-depth and contextualized understanding of your country’s unique family planning journey. This focused group discussion/ in-depth interview will involve exploring FP intersections with gender, vulnerability, socio-cultural paradigms and more specifically discussing the impact of various FP interventions, that have worked, in your country between the years 2000 and 2020.

**Purpose**
The purpose of this study is to understand the determinants and drivers of family planning (FP) uptake with an emphasis on individual factors including social and cultural norms, community dynamics as well as broader political and economic factors within your country context.

The interview should take about 60 to 90 minutes of your time. We want to go over a few points before we begin with the interview questions. First, your participation in this interview is voluntary, and you can choose not to take part in this interview. You do not have to answer any questions if you are not comfortable answering and you can withdraw at any time, without consequence.

With your permission, we would like to record the interview. We will also take notes. Only the research team will have access to recording and reading the notes. Your identity will not be disclosed in any way. Do we have your consent for recording this interview?

Before we begin, please feel free to ask any questions, even during the interview.

**Investigators:**

| **Principal Investigator** |  |
| --- | --- |
| **In-country Lead Investigator** |  |

**Funding:**

This research is funded by the Bill and Melinda Gates Foundation.

**Conflict of Interest:**

All of the team investigators have stated they have no conflicts of interest to declare and will not benefit personally, financially, or in some other way from this study*.*

**Purpose of the Research**:

You are being asked to participate in this research study because you are a member of a community-based group living in this community or government representative/NGO official/national or regional stakeholder. The purpose of this study is to understand the determinants and drivers of family planning use.

Information collected from these interviews will be analyzed and incorporated into a manuscript for publication in a peer-reviewed academic journal.

**Description of the Research**:

The study is being conducted in Sierraleone, Lao PDR and Bolivia. If you consent to be part of the study, you will be asked to participate in one semi-structured interview lasting between one to two hours. The interview will be scheduled at your convenience and will take place in a private area. The interview will take place in person (with all appropriate safeguards against COVID-19 in place), but in some circumstances where an in-person meeting is not possible, a phone interview will be conducted. We will audio record this interview and this recording will be transcribed and analyzed by the research team at the leading in-country collaborating institute. Audio recording is optional, and we can still conduct the interview if you opt to not have the interview recorded.

**If Audio Recorded:**
The interview will be audio recorded. The audio recording will be transcribed after the interview and will be analyzed by the research team. The transcription will be done by a member of the study team. Your name or any other identifying information will not be included during the recording, except your voice. Recordings will be kept for one year after publication.

We may collect basic demographic information about your employment, gender, and age which will be noted in our field notes. These field notes will be kept in a password protected file.

**Potential Harms, Discomforts and Inconveniences:**

This interview will involve the disclosure of your personal opinions and experiences and we may use direct quotes from your interview in publications and reports resulting from this study. While these will not be attributed to you directly in any reporting, the information you provide may potentially identify you to people who may know you. This may possibly have an effect on your interactions within the community.

Discomforts or inconveniences associated with participation may include disruption to your everyday schedule related to participation in the interview. You can choose to take a break before continuing the interview or stop the interview completely. Just let a member of the study team know.

**Audio Recording:**
There is a potential risk of loss of your confidentiality because even though your name will not be part of the transcription, your voice may still be identifiable as your voice. Or if anyone mentions identifiers (e.g., your name), during the recording, this may identify you. When transcribing these recordings, we will not use your name.

**Potential Benefits:**

You will not directly benefit from this study. However, the findings of this study will be able to provide key insights on the determinants and drivers of family planning use that can inform future programmatic activities.

**Confidentiality:**

We will respect your privacy. No information about who you are will be given to anyone or published without your permission, unless required by law.

The audio recordings and written data produced from this study will be stored in a secure location by the leading in-country collaborating institute. Each individual will be assigned a code, and the key linking the code and the name will be kept in a password protected file. All audio recordings and transcripts will be identified with only the code. Recordings will be kept for one year after publication. Only members of the research team will have access to the data. This could include external research team members. Following completion of the research study the data will be kept as long as required then destroyed as required by the leading in-country collaborating institute policy. Published study results or reports will not reveal your identity.

**Participation:**

The interviews will take approximately 1 to 2 hours and will be audio recorded to ensure that the information shared during the interview is accurately documented. It is your choice to take part in this study. You may refuse, and you may stop at any time. If you change your mind about participating in the study, you can do so at any time. You have the option to stop the interview while it is still ongoing and withdraw any information you have provided including interview tapes up until the analysis of study data. These will be destroyed at your request. Your involvement or lack of involvement in this study will not affect your access to health services or employment.

New information may emerge over the course of this study that may affect your decision to participate. If this happens, we will inform you and again ask you if you still wish to participate*.*

**Reimbursement**: You will not be paid or reimbursed for any expenses related to being in this study.

**Consent to Participate in a Research Study**

**Study Title:** Exemplars in Family Planning

By signing this research consent form, I understand and confirm that:

1. All of my questions have been answered,

2. I understand the information within this informed consent form,

3. I understand that I have the right to refuse to take part in or withdraw from this study at any time. My decision will not affect my access to health services or employment.

4. I have been told I will be given a signed and dated copy of this consent form.

5. I agree to take part in this study.

**I consent to participate in this study.**

| Printed Name of Participant |  | Participant signature & date (DD/MM/YYYY) |
| --- | --- | --- |

| Printed Name of person who obtained consent |  | Role of person obtaining consent |  | Signature & date (DD/MM/YYYY) |
| --- | --- | --- | --- | --- |
